# Analysis of the natural radioactivity concentrations of the fine dust samples in Jeju Island, Korea and the annual effective radiation dose by inhalation

**DOI:** 10.1007/s10967-018-5873-6

**Published:** 2018-04-26

**Authors:** Chung Hun Han, Jae Woo Park

**Affiliations:** 1 Institute for Nuclear Science and Technology, Jeju National University, 102 Jejudaehak-ro, Jeju, 63243 Korea; 2 Department of Nuclear & Energy Engineering, Jeju National University, 102 Jejudaehak-ro, Jeju, 63243 Korea

**Keywords:** Fine dust, PM_10_, Natural radioactivity, Jeju

## Abstract

This study analyzed the concentrations of potassium, thorium and uranium of the atmospheric PM_10_ aerosols which were collected at Gosan of Jeju Island during the year of 2014. The mean mass concentration of PM_10_ was 47.31 μg/m^3^. The mean radioactive concentrations of ^40^K, ^232^Th and ^238^U were 7.89, 0.25 and 0.30 μBq/m^3^, respectively. The ^232^Th/^238^U activity concentration ratio of PM_10_ was 0.830. The ^232^Th/^238^U ratio during Asian Dust days is 1.073, which is higher than those in other atmospheric conditions. The concentration ratio of ^232^Th/^238^U was 0.902 in China continent.

## Introduction

Gamma radiation emitted from naturally occurring radioisotopes, such as ^40^K and the radionuclides from the ^232^Th and ^238^U series and their decay products, which exist at trace levels in all ground formations, represents the main external source of irradiation to the human body [1–3]. The human beings are exposed mainly to natural sources of radioactivity. More than 80% of the radiation dose received by mankind is due to natural radiation sources [1]. Natural radioactivity is associated mainly to primordial radionuclides, including the isotopes ^40^K and the progeny of the ^238^U and ^232^Th decay series.

The annual worldwide per caput effective radiation dose from natural source is determined by adding the various components. The annual global per caput effective dose due to natural radiation sources is 2.4 mSv. However, the range of individual doses is wide. In any large population about 65% would be expected to have annual effective doses between 1 and 3 mSv, about 25% of the population would have annual effective doses less than 1 mSv and 10% would have annual effective doses greater than 3 mSv [1].

Numerous studies all over the world have been conducted to determine the activity of these radioisotopes in soils and rocks to estimate the external gamma-radiation dose to the public [1]. Several studies have been conducted in the countries of the Asian continent, to determine the activity concentrations of the naturally occurring radionuclides in soil and rock samples [4–10]. In most of these studies the external radiation dose was calculated. Internal radiation dose from ingestion, tobacco and building materials were also studied [11–14]. However, internal dose due to inhalation of fine aerosol particles in the surrounded Korean peninsula are not found in the literature.

The Gosan area in Jeju Island, South Korea serves as a natural background site for characterizing the air pollution in Korean peninsula since the atmosphere in the area is relatively very little affected by artificial airborne matters. Chemical composition analyses have usually been conducted for the atmospheric aerosols collected at the site. In this study, we have measured, using the Inductively Coupled Plasma-Dynamic Reaction Cell-Mass Spectrometer (ICP-DRC-MS), radioactivity concentrations of ^40^K, ^232^Th and ^238^U contained in the atmospheric PM_10_ aerosols which were collected at the Gosan during the year of 2014.

## Experimental

### Air sampling locations

**Fig. 1:**
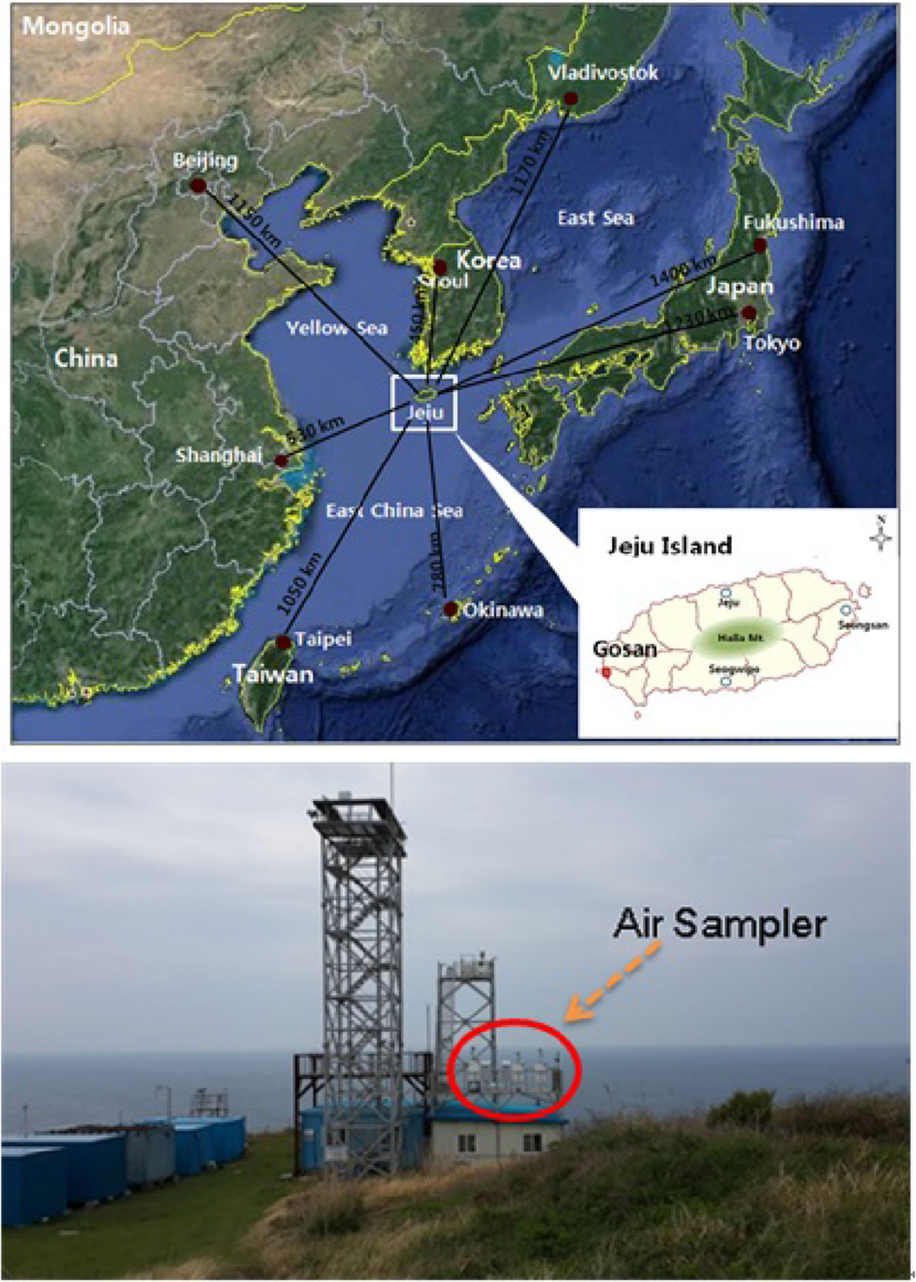
Location of air sampling Gosan site in Jeju Island, Korea

Air sampling for PM_10_ aerosols was conducted at the Gosan (33°1′N, 126°10′E), which is located at the western edge of Jeju Island facing the Asian continent (Fig. 1) [15]. Jeju Island, Korea, is located at the boundary of the East China Sea and the Yellow Sea and is surrounded by mainland China, the Korean peninsula, and Japan. The Gosan sampling site is located on the hill of 72 m above sea level and is isolated from residential areas on the island. In order to understand physicochemical and radioactive properties of anthropogenic aerosols under Asian continental outflow, several international experiments have been conducted at the Gosan site, such as ACE-Asia (Aerosol Characterization Experiment-Asia) [16] and ABC-EAREX 2005 (Atmospheric Brown Cloud-East Asia Regional Experiment 2005) [17]. To understand the link between physical and chemical properties of aerosols transported from the Asian continent and regional climate change, we need to investigate the sources and formation mechanism of secondary aerosols. In addition, contributions of local effects on the Gosan site aerosols should be qualitatively and quantitatively evaluated [18].

### Collection of atmospheric aerosols

The PM_10_ samples have been collected using PMS-103 (APM Engineering, PMS-103, Korea), which is an automatic system with Teflon filter (Pall Corporation, ZeflourTM, 47 mm/2.0 µm, USA) at 24 h basis with every 3 day intervals during the year of 2014. The air flow rate was kept to about 16.7 L/min, and total air flow was calculated from the flow rate and running time with MFC (mass flow controller). Quality assurance and quality control procedures were followed, including calibration of the sampler flow rates, collecting field and trip blanks, inspection and conditioning of the filter media before and after use.

### Sample analysis

For obtaining accurate concentrations of the radionuclides of interest, complete digestion is required before ICP-MS. The addition of hydrofluoric acid strongly influences the recovery of the microwave acid digestion of environmental samples because it breaks down silicates and minerals better than other acid combinations [19]. However, due to the problems in glassware and torch damage of ICP-MS as well as toxicity of HF, the acid combination HNO_3_/HCl was used for the digestion process [19].

The PM_10_ aerosols were decomposed with acids using a START D microwave digestion system (Milestone, Italy). The aerosol filters were put in a perfluoroalkoxy Teflon vessel of the microwave digestion system together with 10 mL acid solution (5.55% HNO_3_/16.75% HCl). This vessel was heated at 180 °C for 15 min with 1000 W microwave radiation to digest the PM_10_ aerosols. The decomposed solution was transferred through 0.45 μm PVDF syringe filter (Whatman) into a 25 mL volumetric flask, and the volume was adjusted to 25 mL with 5 mL acid solution (3% HNO_3_/8% HCl) and ultrapure water [20]. The number of elements determined by ICP-DRC-MS (Perkin Elmer, ELAN DRC-II, USA) instruments was 3 species such as ^39^K, ^232^Th and ^238^U. The instrumental conditions of ICP-DRC-MS were as follows: 40 MHz RF frequency, 1500 W RF power, 15.0 L min^−1^ coolant, 0.9–1.05 L min^−1^ Ar flow, 1.2 L min^−1^ auxiliary. The DRC gas was used as the ammonia. The instrumental detection limits (IDL) of ^39^K, ^232^Th and ^238^U were 27.12 μg L^−1^, 2.11 ng L^−1^ and 2.89 ng L^−1^, respectively (Table 1).

**Table 1:** Instrumental detection limit (IDL) and coefficient of variation (CV) for ICP-DRC-MS (n = 7)

Species	^39^K (μg L^−1^)	^232^Th (ng L^−1^)	^238^U (ng L^−1^)
IDL	27.12	2.11	2.89
CV (%)	2.24	1.40	1.54

### Isotope activity concentration

Potassium, thorium and uranium were measured as trace elements in PM_10_ aerosol by ICP-DRC-MS. The radioactivity concentrations of the isotopes ^40^K, ^232^Th and ^238^U in the collected PM_10_ were calculated using the following Eq. [1] [21]:

1 $$A_{\text{i}} = \frac{\ln 2}{{T_{1/2} }} \times \frac{{\rho_{\text{i}} \times m_{\text{e}} }}{{M_{\text{i}} }} \times N$$

where *A*_i_, *T*_1/2_, *ρ*_i_, *m*_e_, *M*_i_, and *N* are the radioactivity concentration (Bq m^−3^), half-life time (s) of isotope *i* (^40^K: 1.28 × 10^9^ years, ^238^U: 4.468 × 10^9^ years, ^232^Th: 1.405 × 10^10^ years), isotopic ratio (natural abundance) of isotope *i*, the mass concentration of element e corresponding to isotope i (g m^−3^), atomic mass (g mol^−1^), and the Avogadro’s number (6.022 × 10^23^ mol^−1^), respectively. The isotopic ratios for ^40^K, ^238^U and ^232^Th were 0.000117, 0.99275 and 1.0, respectively, based on the International Union of Pure and Applied Chemistry (IUPAC) report 2009 [22].

### Inhalation annual effective dose

The inhalation annual effective radiation dose (*E*_i_) due to PM_10_ was calculated using the following Eq. [2] [1]:

2 $$E_{\text{i}} = A_{\text{i}} \times B \times d_{\text{i}} \left( {1 \, - F_{0} + F_{0} F_{\text{r}} } \right)$$

where *A*_i_ is the integrated activity concentration of radionuclide *i* associated with PM_10_ in outdoor air (Bq m^−3^), *B* is the breathing rate (m^3^ year^−1^), *d*_i_ is the committed dose per unit intake from inhalation or effective dose coefficient (Sv Bq^−1^), *F*_0_ is the indoor occupancy factor and *F*_r_ is the ratio of indoor to outdoor air concentration.

The annual effective radiation dose due to inhalation of PM_10_ was calculated for the six age groups identified by the International Commission on Radiological Protection (ICRP), namely 3 months, 1, 5, 10, 15 years and adults. For each of the age groups, the corresponding breathing rate and indoor occupancy factor [23], and effective dose coefficient for inhalation [24] were used. These values are summarized in Tables 2 and 3. The default modes of absorption for the isotopes were used. For the effective dose coefficient, the activity median aerodynamic diameter (AMAD) was assumed to be 1 μm. The ratio of indoor to outdoor air concentration (*F*_r_) was assumed to be 0.3 [1, 25, 26].

**Table 2:** Breathing rate and indoor occupancy factor values used for calculating the inhalation annual effective radiation dose due to inhalation of PM_10_

Age group	3 months	1 years	5 years	10 years	15 years		Adult	
					Male	Female	Male	Female
Breathing rate, *B* (m^3^ day^−1^)	2.8	5.1	8.8	15.2	20.1	15.8	22.2	18.2
Indoor occupancy factor, *F*_0_	1.00	0.96	0.88	0.88	0.88	0.92	0.92	0.92

**Table 3:** Effective dose coefficient for inhalation *d*_i_ (μSv Bq^−1^) used for calculating the inhalation annual effective radiation dose due to inhalation of PM_10_

Nuclide	Type	3 months	1 years	5 years	10 years	15 years	Adult
^40^K	F	0.024	0.017	0.0075	0.0045	0.0025	0.0021
^238^U	F	1.9	1.3	0.82	0.73	0.74	0.50
	M	12	9.4	5.9	4.0	3.4	2.9
	S	29	25	16	10	8.7	8.0
^232^Th	F	230	220	160	130	120	110
	M	83	81	63	50	47	45
	S	54	50	37	26	25	25

Type F: deposited materials that are readily absorbed into blood from the respiratory; type M: deposited materials that have intermediate rates of absorption into blood from the respiratory tract; type S: deposited materials that are relatively insoluble in the respiratory tract

## Results and discussion

### ^40^K, ^232^Th and ^238^U radioactivity concentration in PM_10_

As an alternative method, we have measured, using the ICP-DRC-MS, radioactivity concentrations of ^40^K, ^232^Th and ^238^U contained in the atmospheric PM_10_ aerosols which were collected at the Gosan during the year of 2014. A total of 115 samples have been analyzed, of which 5 samples are those collected during Asian Dust days, 47 samples collected during normal weather days (no-event days), and the remaining samples collected during the days of haze (6 samples) and fog-mist (57 samples).

The mean mass concentration of PM_10_ was 47.31 μg/m^3^. During the study period, the mean concentrations of ^40^K, ^232^Th and ^238^U were 0.56, 1.02 and 0.53 mg/kg-dust, respectively. Furthermore, during the study period, the activity concentrations of the radionuclides ^232^Th and ^238^U were 0–16.86 (mean 4.16 ± 2.98 Bq/kg-dust) and 0–62.07 (mean 6.48 ± 7.43 Bq/kg-dust), respectively. They are lower than the world averages for activity concentrations of ^232^Th and ^238^U, which are 30 and 35 Bq/kg-dust, respectively [27]. The mean active concentrations of ^40^K, ^232^Th and ^238^U during normal days are 7.89 ± 10.89, 0.25 ± 0.37 and 0.30 ± 0.35 μBq/m^3^, respectively. Furthermore, they are lower than the world averages for the mean atmospheric activity concentrations of ^232^Th and ^238^U associated with dust, which are 0.5 and 1.0 μBq/m^3^, respectively [27]. It is worth noting that the world average is based on total suspended particulate. The low atmospheric activity concentration of ^232^Th and ^238^U might be attributed to the low atmospheric PM_10_ in Gosan site as compared to many of the world cities. The ^232^Th/^238^U activity concentration ratio of PM_10_ was 0.830.

### Activity concentrations by atmospheric phenomenon

The radioactivity concentrations of those isotopes were analyzed by atmospheric phenomenon (Asian Dust, Haze, Fog-Mist and Non-Event). During Asian Dust periods (5 samples), the mean concentrations of ^40^K, ^232^Th and ^238^U were 1.24, 2.81 and 0.87 mg/kg-dust, respectively. Furthermore, during the Asian Dust period, the activity concentrations of the radionuclides ^232^Th and ^238^U were 7.44–16.86 (mean 11.38 ± 3.83 Bq//kg-dust) and 9.19–13.45 (mean 10.71 ± 1.72 Bq/kg-dust), respectively. Table 4 shows also that the mean atmospheric activity concentration of ^232^Th and ^238^U of Asian Dust periods associated with PM_10_ was 1.47 ± 0.72 and 1.37 ± 0.55 μBq/m^3^, exceeding the revised world reference values. And these were highly as 6.94, 8.57 and 7.05 times, respectively, compared to the non-event periods (47 samples). The ^232^Th/^238^U ratio of Asian Dust was 1.073, which was higher than those of other atmospheric phenomenon (0.707–0.882) (Table 5).

**Table 4:** The associated ^40^K, ^238^U and ^232^Th concentrations of airborne PM_10_ by atmospheric phenomenon at Gosan site in Jeju Island, 2014

Atmospheric phenomenon	^40^K		^232^Th		^238^U	
	mg/kg-dust	μBq/m^3^	mg/kg-dust	μBq/m^3^	mg/kg-dust	μBq/m^3^
Asian Dust (n = 5)	1.24 ± 0.64	40.89 ± 23.20	2.81 ± 0.94	1.47 ± 0.72	0.87 ± 0.14	1.37 ± 0.55
Haze (n = 6)	0.61 ± 0.26	18.29 ± 17.73	1.25 ± 0.50	0.50 ± 0.40	0.65 ± 0.32	0.50 ± 0.44
Fog-Mist (n = 57)	0.53 ± 0.33	5.58 ± 6.24	0.94 ± 0.66	0.17 ± 0.23	0.62 ± 0.80	0.24 ± 0.24
Non-Event (n = 47)	0.51 ± 0.36	5.85 ± 4.67	0.91 ± 0.57	0.17 ± 0.15	0.37 ± 0.25	0.19 ± 0.16
All (n = 115)	0.56 ± 0.36	7.89 ± 10.89	1.03 ± 0.73	0.25 ± 0.37	0.53 ± 0.60	0.30 ± 0.35

**Table 5:** The activity ratio of ^40^K, ^232^Th and ^238^U activity concentrations (μBq/m^3^) by atmospheric phenomenon at Gosan site in Jeju Island, 2014

Atmospheric phenomenon	^232^Th/^40^K	^238^U/^40^K	^232^Th/^238^U
Asian Dust	0.036	0.033	1.073
Haze	0.028	0.039	0.707
Fog-Mist	0.031	0.043	0.715
Non-Event	0.029	0.033	0.882
All	0.031	0.037	0.830

### Inflow pathways of air mass

**Fig. 2:**
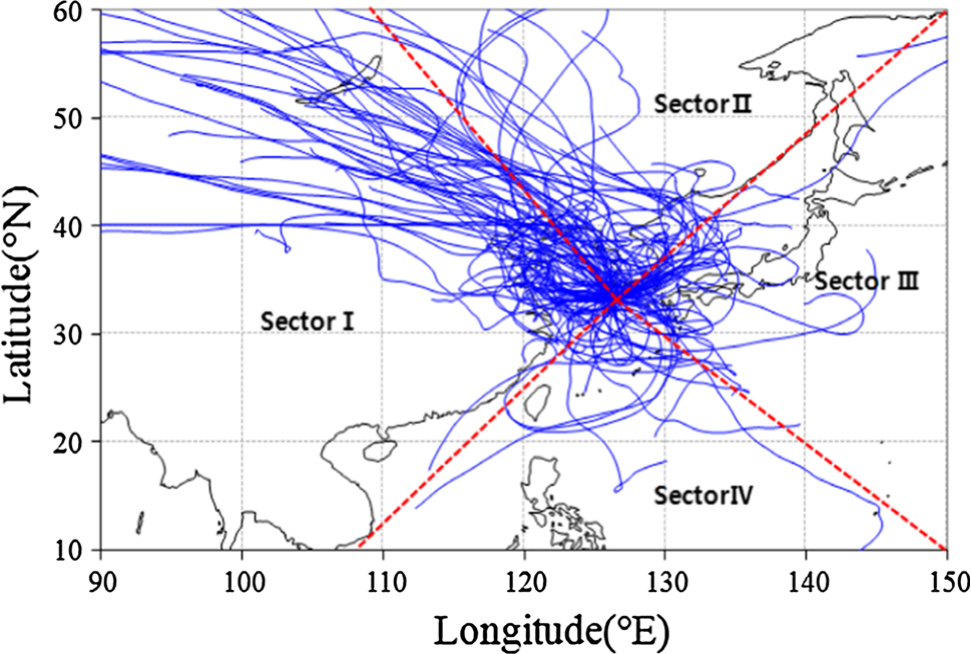
5-Day backward trajectories at the Gosan Site of Jeju Island during the study period

We are analyzing five-day backward trajectories of the air inflow into the Gosan site using the HYSPLIT4 (HYbrid Single Particle Lagrangian Integrated Trajectory) model of National Oceanic and Atmospheric Administration (NOAA, USA) [28]. The frequency of air inflow into the Gosan site is 34.84% from Sector I (China continent), 22.58% from Sector II (Korean peninsula), 11.61% from Sector III (Japan) and 5.16% from Sector IV (North Pacific Ocean) during normal days. As a result of analyzing the air volume, the concentration ratio of ^232^Th/^40^K was 0.033 and ^238^U/^40^K was 0.036 in Sector 1 (Table 6). The concentration ratio of ^232^Th/^238^U in this sector was measured as 0.902. For sector 2, ^238^U/^40^K was 0.041 and ^232^Th/^40^K was 0.031. And ^232^Th/^238^U in this sector was measured as 0.750. During Asian dust days, the air inflow is dominated from Sector I (Fig. 2).

**Table 6:** Activity concentrations and ratio of nuclides according to the sector

Sector	^40^K (μBq/m^3^)	^232^Th (μBq/m^3^)	^238^U (μBq/m^3^)	^232^Th/^40^K	^238^U/^40^K	^232^Th/^238^U
I	11.38 ± 14.24	0.37 ± 0.48	0.41 ± 0.43	0.033	0.036	0.902
II	5.88 ± 5.45	0.18 ± 0.17	0.24 ± 0.24	0.031	0.041	0.750
III	4.11 ± 4.84	0.10 ± 0.12	0.14 ± 0.15	0.024	0.034	0.714
IV	2.10 ± 1.40	0.03 ± 0.02	0.09 ± 0.16	0.014	0.043	0.333

### Inhalation annual effective dose

**Fig. 3:**
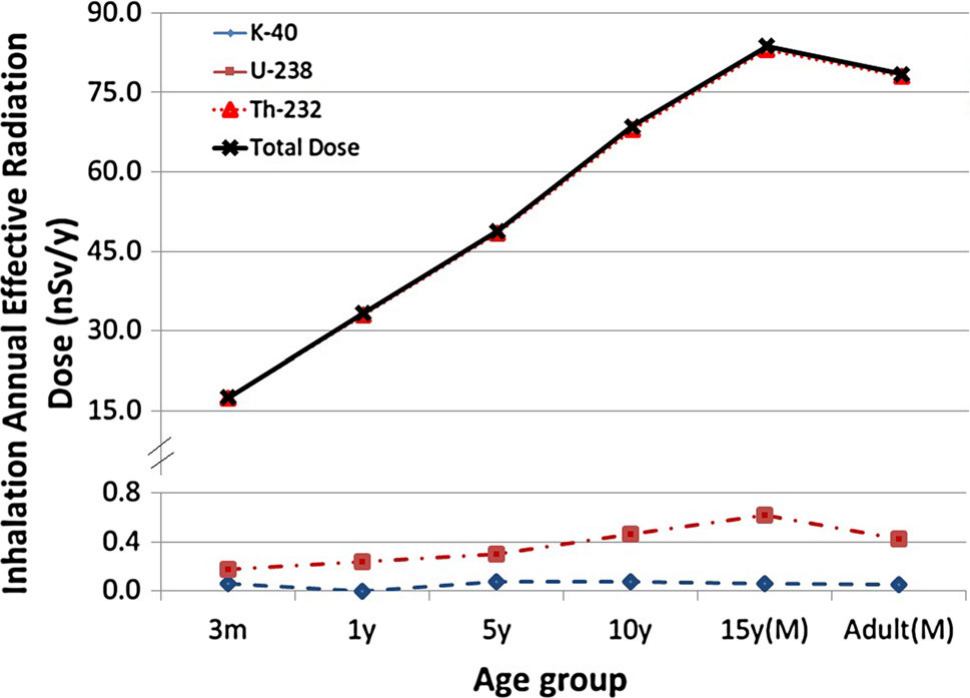
Annual effective dose to various age groups in Jeju Island from inhalation ^40^K, ^238^U, and ^232^Th in PM_10_ aerosols (F type, Male)

Isotopes activity concentrations in air (μBq/m^3^) presented in Table 4 were used in the calculation of inhalation annual effective radiation dose (*E*_i_) to various age groups of the public. Table 7 and Fig. 3 show the results of these calculations. For instance, *E*_Total_ was 17.56 nSv/year to the infants (< 1 year), while it was 78.45 nSv/year to the adults (male), depending on the age group. Because of the variations in air breathing rate, the total annual dose due to the natural radioactivity in airborne PM_10_ increases for the older age groups. These values are higher than the corresponding values calculated from the data presented in UNSCEAR 2000 [27]. It is obvious that ^232^Th is the main contributor to the inhalation annual effective dose. ^232^Th was found to be responsible for the total dose. On the other hand, ^40^K was found to slightly contribute to the total dose.

**Table 7:** The average inhalation annual effective dose (nSv/year) to various age groups (default mode F) of the public in Gosan site from ^40^K, ^238^U and ^232^Th associated with airborne PM_10_

Radionuclide	3 months	1 years	5 years	10 years	15 years		Adult	
					Male	Female	Male	Female
^40^K	0.06	0.08	0.07	0.08	0.06	0.03	0.05	0.04
^238^U	0.17	0.24	0.30	0.46	0.62	0.45	0.43	0.35
^232^Th	17.33	33.01	48.49	68.05	83.07	60.54	77.97	63.92
Total	17.56	33.32	48.86	68.59	83.74	61.02	78.45	64.31

## Conclusions

The atmospheric PM_10_ aerosols (115 samples) were collected at Gosan of Jeju Island, which is one of the natural background sites of Korea, during the year of 2014. This study analyzed using ICP-DRC-MS the concentrations of potassium, thorium and uranium, and evaluated the annual effective dose by breathing from the results. The mean mass concentration of PM_10_ was 47.308 μg/m^3^. The mean radioactive concentrations of ^40^K, ^232^Th and ^238^U were 7.89, 0.25 and 0.30 μBq/m^3^, respectively. The ^232^Th/^238^U activity concentration ratio of PM_10_ was 0.830. The ^232^Th/^238^U ratio during Asian Dust days is 1.073, which is higher than those in other atmospheric conditions. During Asian Dust days, the air inflow is dominated from Sector I (China continent). The concentration ratio of ^232^Th/^238^U was 0.902 in Sector 1. In this study, the ratio of each nuclide was compared according to the inflow route. As a result, it was confirmed that the ratio of each nuclide was slightly different according to the inflow route. It is expected that this will be a preliminary data for analyzing the sources and various weather phenomena of fine dust on the peninsula. Jeju Island with less pollution source and low population density is also one of the best places as a background area in Asia. It is judged that the results become a preliminary data on the impact of fine dust from China, which has been recently intensified, on the Korean Peninsula. Therefore, it is judged as necessary to observe such Asian Dust phenomenon in the long term through collecting a large number of the PM_10_ aerosol filters.
